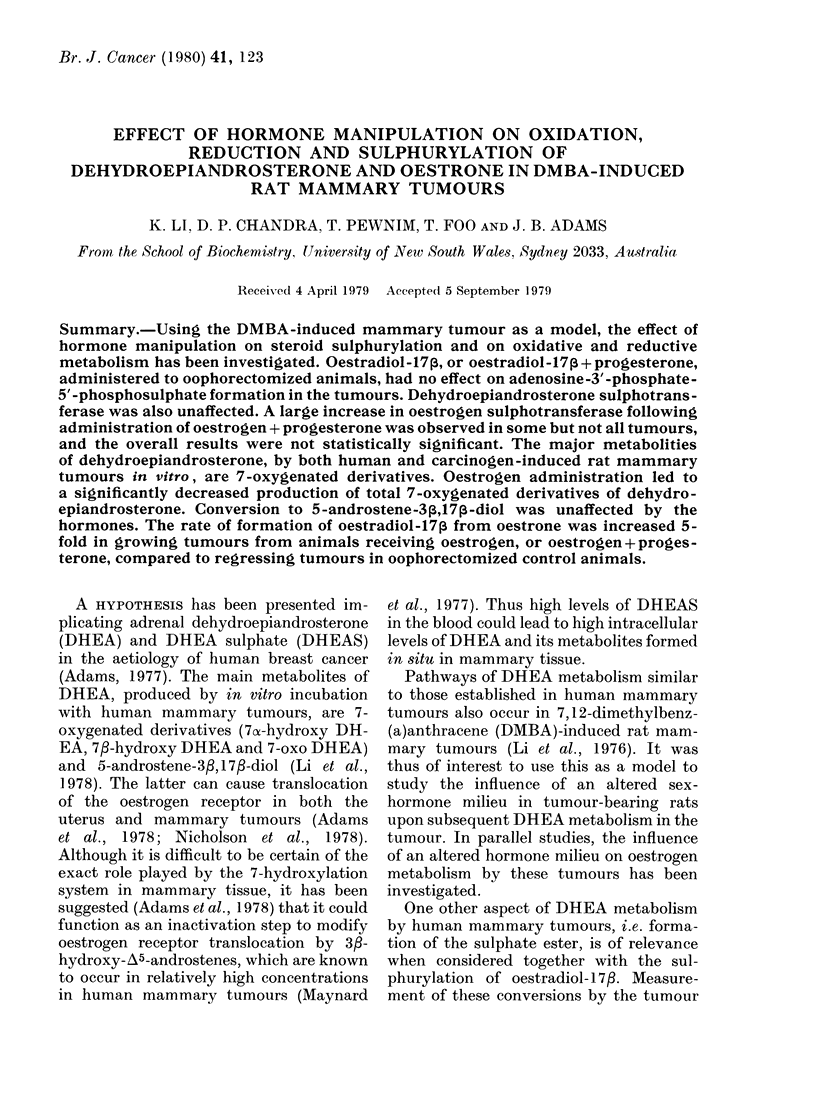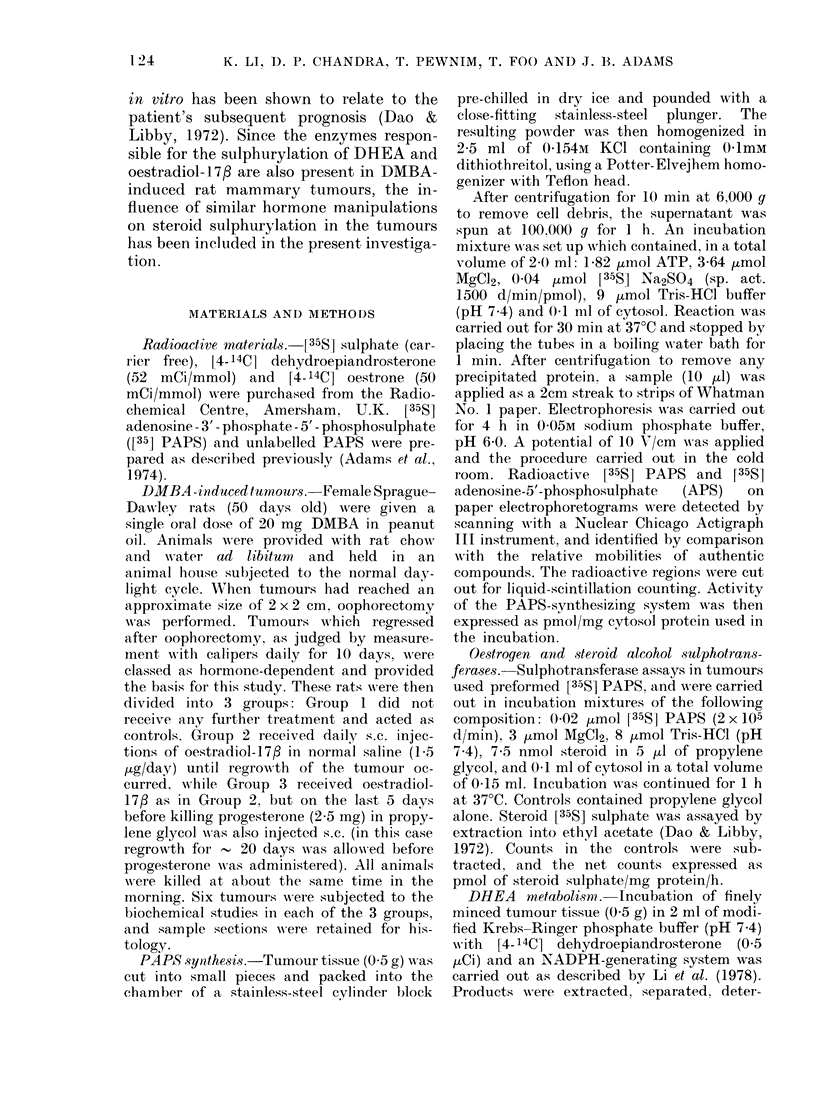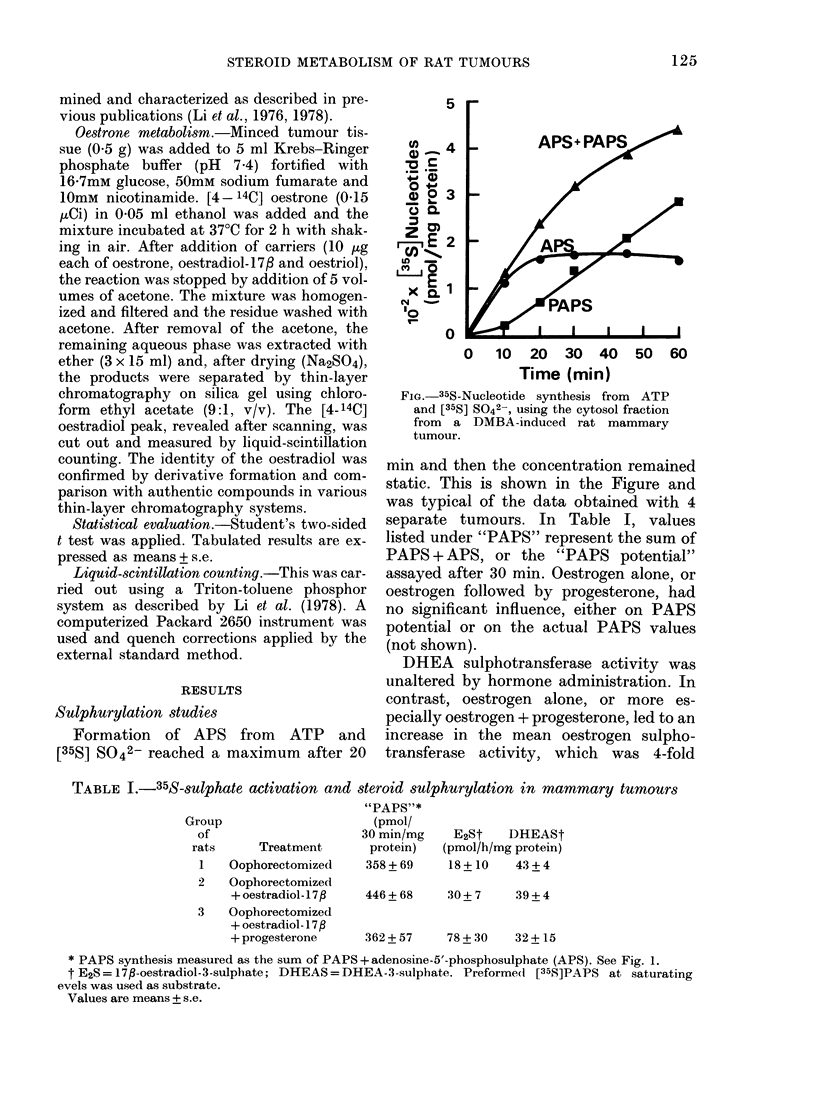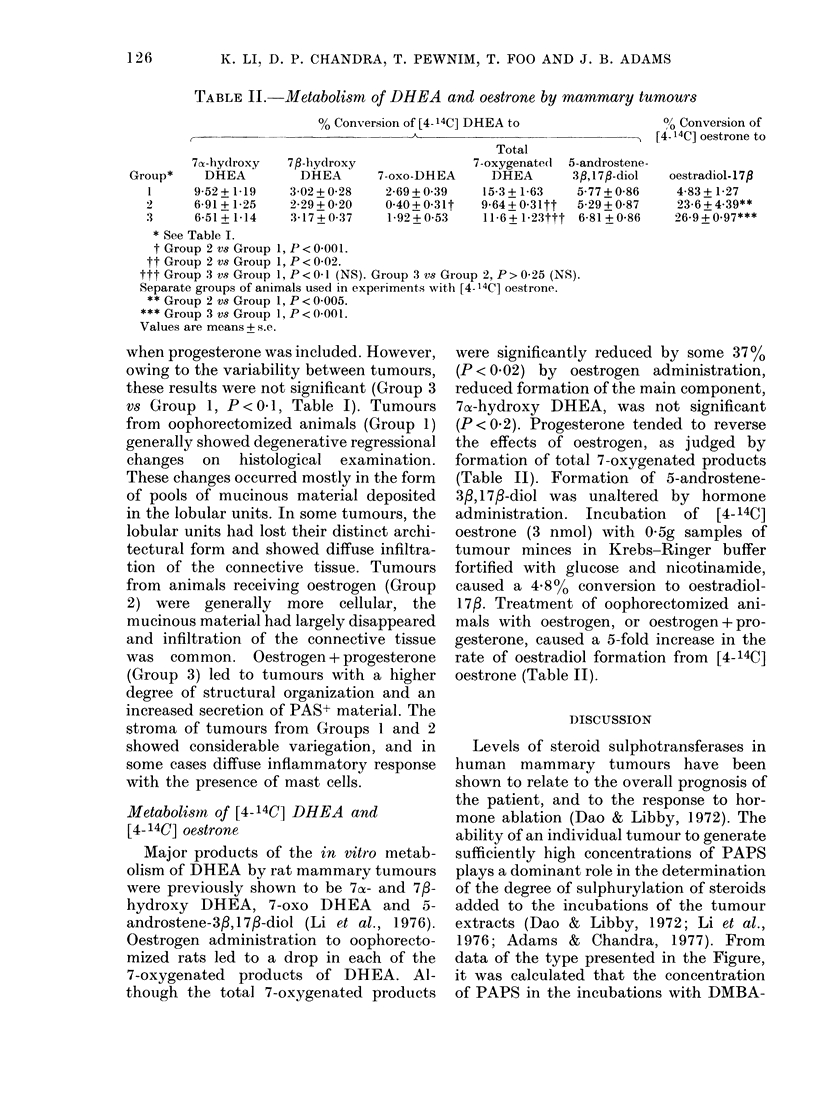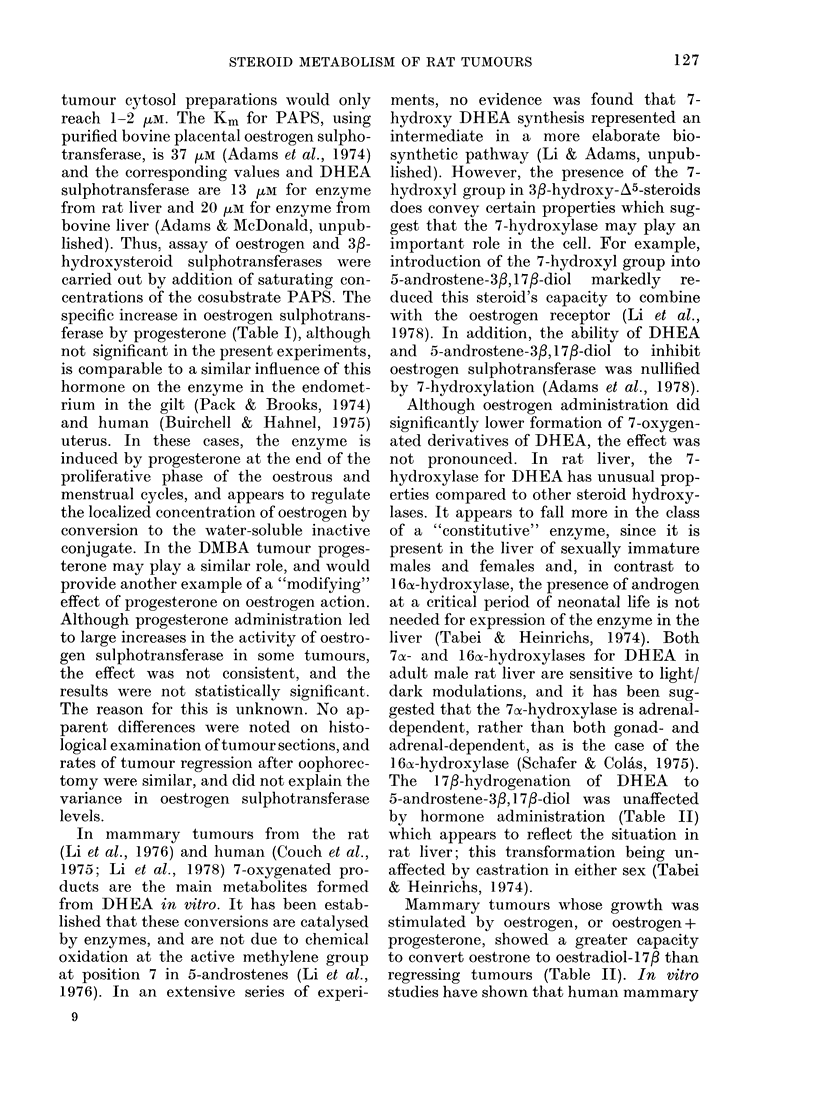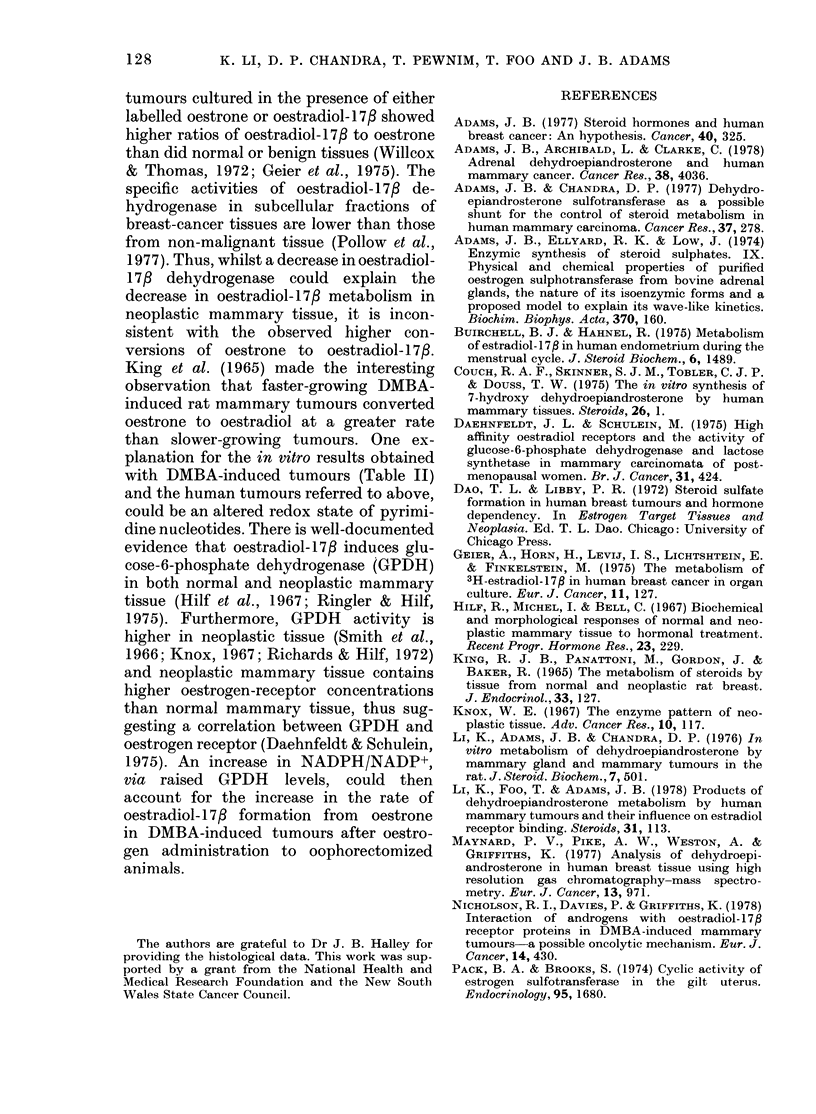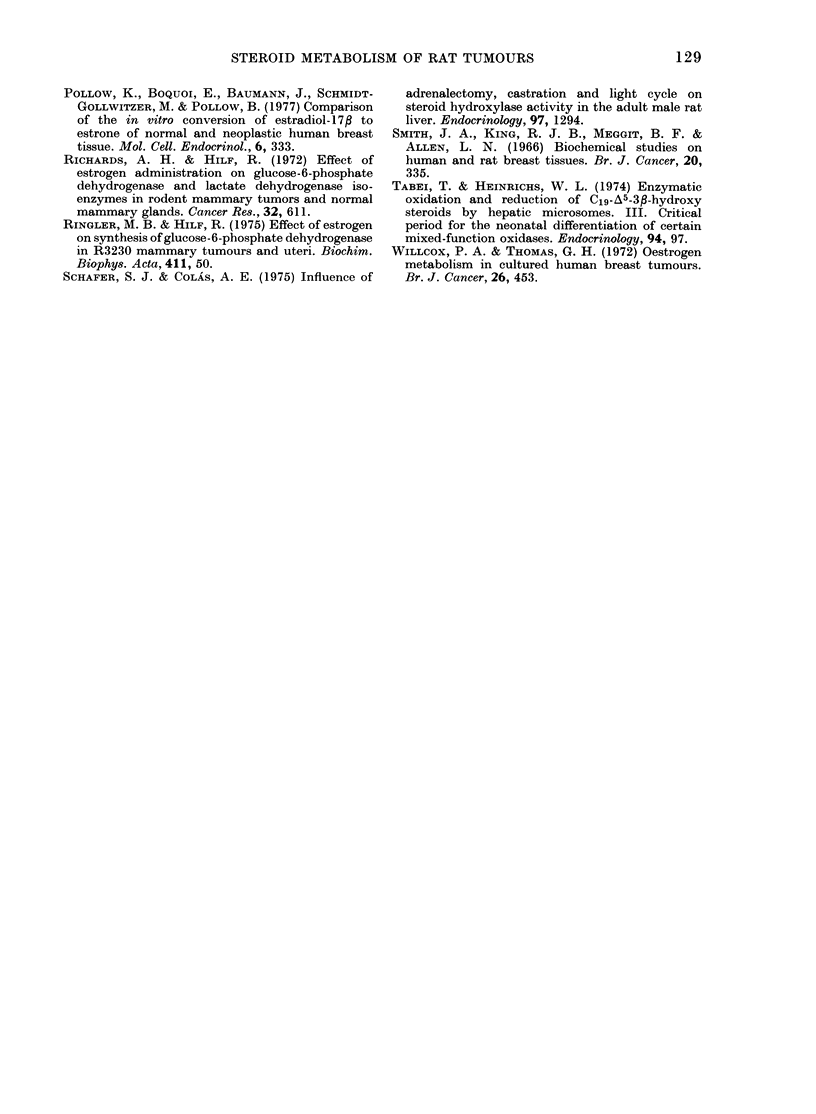# Effect of hormone manipulation on oxidation, reduction and sulphurylation of dehydroepiandrosterone and oestrone in DMBA-induced rat mammary tumours.

**DOI:** 10.1038/bjc.1980.14

**Published:** 1980-01

**Authors:** K. Li, D. P. Chandra, T. Pewnim, T. Foo, J. B. Adams

## Abstract

Using the DMBA-induced mammary tumour as a model, the effect of hormone manipulation on steroid sulphurylation and on oxidative and reductive metabolism has been investigated. Oestradiol-17 beta, or oestradiol-17 beta + progesterone, administered to oophorectomized animals, had no effect on adenosine-3'-phosphate-5'-phosphosulphate formation in the tumours. Dehydroepiandrosterone sulphotransferase was also unaffected. A large increase in oestrogen sulphotransferase following administration of oestrogen + progesterone was observed in some but not all tumours, and the overall results were not statically significant. The major metabolities of dehydroepiandrosterone, by both human and carcinogen-induced rat mammary tumours in vitro, are 7-oxygenated derivatives. Oestrogen administration led to a significantly decreased production of total 7-oxygenated derivatives of dehydroepiandrosterone. Conversion to 5-androstene-3 beta, 17 beta-diol was unaffected by the hormones. The rate of formation of oestradiol-17 beta from oestrone was increased 5-fold in growing tumours from animals receiving oestrogen, or oestrogen + progesterone, compared to regressing tumours in oophorectomized control animals.


*Br. J. Cancer* (1980) **41**, 123

### EFFECT OF HORMONE MANIPULATION ON OXIDATION, REDUCTION AND SULPHURYLATION OF DEHYDROEPIANDROSTERONE AND OESTRONE IN DMBA-INDUCED RAT MAMMARY TUMOURS

K. LI, D. P. CHANDRA, T. PEWNIM, T. FOO AND J. B. ADAMS

*From the School of Biochemistry, University of New South Wales, Sydney* 2033, *Australia*

Received 4 April 1979 Accepted 5 September 1979

**Summary.—Using the DMBA-induced mammary tumour as a model, the effect of hormone manipulation on steroid sulphurylation and on oxidative and reductive metabolism has been investigated. Oestradiol-17β, or oestradiol-17β + progesterone, administered to oophorectomized animals, had no effect on adenosine-3′-phosphate-5′-phosphosulphate formation in the tumours. Dehydroepiandrosterone sulphotransferase was also unaffected. A large increase in oestrogen sulphotransferase following administration of oestrogen + progesterone was observed in some but not all tumours, and the overall results were not statistically significant. The major metabolities of dehydroepiandrosterone, by both human and carcinogen-induced rat mammary tumours *in vitro*, are 7-oxygenated derivatives. Oestrogen administration led to a significantly decreased production of total 7-oxygenated derivatives of dehydroepiandrosterone. Conversion to 5-androstene-3β,17β-diol was unaffected by the hormones. The rate of formation of oestradiol-17β from oestrone was increased 5-fold in growing tumours from animals receiving oestrogen, or oestrogen + progesterone, compared to regressing tumours in oophorectomized control animals.**

A HYPOTHESIS has been presented implicating adrenal dehydroepiandrosterone (DHEA) and DHEA sulphate (DHEAS) in the aetiology of human breast cancer (Adams, 1977). The main metabolites of DHEA, produced by *in vitro* incubation with human mammary tumours, are 7-oxygenated derivatives (7α-hydroxy DHEA, 7β-hydroxy DHEA and 7-oxo DHEA) and 5-androstene-3β,17β-diol (Li *et al.*, 1978). The latter can cause translocation of the oestrogen receptor in both the uterus and mammary tumours (Adams *et al.*, 1978; Nicholson *et al.*, 1978). Although it is difficult to be certain of the exact role played by the 7-hydroxylation system in mammary tissue, it has been suggested (Adams *et al.*, 1978) that it could function as an inactivation step to modify oestrogen receptor translocation by 3β-hydroxy-$\Delta^5$-androstenes, which are known to occur in relatively high concentrations in human mammary tumours (Maynard *et al.*, 1977). Thus high levels of DHEAS in the blood could lead to high intracellular levels of DHEA and its metabolites formed *in situ* in mammary tissue.

Pathways of DHEA metabolism similar to those established in human mammary tumours also occur in 7,12-dimethylbenz-(a)anthracene (DMBA)-induced rat mammary tumours (Li *et al.*, 1976). It was thus of interest to use this as a model to study the influence of an altered sex-hormone milieu in tumour-bearing rats upon subsequent DHEA metabolism in the tumour. In parallel studies, the influence of an altered hormone milieu on oestrogen metabolism by these tumours has been investigated.

One other aspect of DHEA metabolism by human mammary tumours, *i.e.* formation of the sulphate ester, is of relevance when considered together with the sulphurylation of oestradiol-17β. Measurement of these conversions by the tumour

*in vitro* has been shown to relate to the patient's subsequent prognosis (Dao & Libby, 1972). Since the enzymes responsible for the sulphurylation of DHEA and oestradiol-17$\beta$ are also present in DMBA-induced rat mammary tumours, the influence of similar hormone manipulations on steroid sulphurylation in the tumours has been included in the present investigation.

### MATERIALS AND METHODS

*Radioactive materials.*—[$^{35}$S] sulphate (carrier free), [4-$^{14}$C] dehydroepiandrosterone (52 mCi/mmol) and [4-$^{14}$C] oestrone (50 mCi/mmol) were purchased from the Radiochemical Centre, Amersham, U.K. [$^{35}$S] adenosine-3′-phosphate-5′-phosphosulphate ([$^{35}$] PAPS) and unlabelled PAPS were prepared as described previously (Adams *et al.*, 1974).

*DMBA-induced tumours.*—Female Sprague–Dawley rats (50 days old) were given a single oral dose of 20 mg DMBA in peanut oil. Animals were provided with rat chow and water *ad libitum* and held in an animal house subjected to the normal daylight cycle. When tumours had reached an approximate size of 2 × 2 cm, oophorectomy was performed. Tumours which regressed after oophorectomy, as judged by measurement with calipers daily for 10 days, were classed as hormone-dependent and provided the basis for this study. These rats were then divided into 3 groups: Group 1 did not receive any further treatment and acted as controls, Group 2 received daily s.c. injections of oestradiol-17$\beta$ in normal saline (1·5 $\mu$g/day) until regrowth of the tumour occurred, while Group 3 received oestradiol-17$\beta$ as in Group 2, but on the last 5 days before killing progesterone (2·5 mg) in propylene glycol was also injected s.c. (in this case regrowth for $\sim$ 20 days was allowed before progesterone was administered). All animals were killed at about the same time in the morning. Six tumours were subjected to the biochemical studies in each of the 3 groups, and sample sections were retained for histology.

*PAPS synthesis.*—Tumour tissue (0·5 g) was cut into small pieces and packed into the chamber of a stainless-steel cylinder block pre-chilled in dry ice and pounded with a close-fitting stainless-steel plunger. The resulting powder was then homogenized in 2·5 ml of 0·154M KCl containing 0·1mM dithiothreitol, using a Potter-Elvejhem homogenizer with Teflon head.

After centrifugation for 10 min at 6,000 *g* to remove cell debris, the supernatant was spun at 100,000 *g* for 1 h. An incubation mixture was set up which contained, in a total volume of 2·0 ml: 1·82 $\mu$mol ATP, 3·64 $\mu$mol $MgCl_2$, 0·04 $\mu$mol [$^{35}$S] $Na_2SO_4$ (sp. act. 1500 d/min/pmol), 9 $\mu$mol Tris-HCl buffer (pH 7·4) and 0·1 ml of cytosol. Reaction was carried out for 30 min at 37°C and stopped by placing the tubes in a boiling water bath for 1 min. After centrifugation to remove any precipitated protein, a sample (10 $\mu$l) was applied as a 2cm streak to strips of Whatman No. 1 paper. Electrophoresis was carried out for 4 h in 0·05M sodium phosphate buffer, pH 6·0. A potential of 10 V/cm was applied and the procedure carried out in the cold room. Radioactive [$^{35}$S] PAPS and [$^{35}$S] adenosine-5′-phosphosulphate (APS) on paper electrophoretograms were detected by scanning with a Nuclear Chicago Actigraph III instrument, and identified by comparison with the relative mobilities of authentic compounds. The radioactive regions were cut out for liquid-scintillation counting. Activity of the PAPS-synthesizing system was then expressed as pmol/mg cytosol protein used in the incubation.

*Oestrogen and steroid alcohol sulphotransferases.*—Sulphotransferase assays in tumours used preformed [$^{35}$S] PAPS, and were carried out in incubation mixtures of the following composition: 0·02 $\mu$mol [$^{35}$S] PAPS ($2 \times 10^5$ d/min), 3 $\mu$mol $MgCl_2$, 8 $\mu$mol Tris-HCl (pH 7·4), 7·5 nmol steroid in 5 $\mu$l of propylene glycol, and 0·1 ml of cytosol in a total volume of 0·15 ml. Incubation was continued for 1 h at 37°C. Controls contained propylene glycol alone. Steroid [$^{35}$S] sulphate was assayed by extraction into ethyl acetate (Dao & Libby, 1972). Counts in the controls were subtracted, and the net counts expressed as pmol of steroid sulphate/mg protein/h.

*DHEA metabolism.*—Incubation of finely minced tumour tissue (0·5 g) in 2 ml of modified Krebs–Ringer phosphate buffer (pH 7·4) with [4-$^{14}$C] dehydroepiandrosterone (0·5 $\mu$Ci) and an NADPH-generating system was carried out as described by Li *et al.* (1978). Products were extracted, separated, determined and characterized as described in previous publications (Li *et al.*, 1976, 1978).

*Oestrone metabolism.*—Minced tumour tissue (0·5 g) was added to 5 ml Krebs–Ringer phosphate buffer (pH 7·4) fortified with 16·7mM glucose, 50mM sodium fumarate and 10mM nicotinamide. [4 – $^{14}$C] oestrone (0·15 μCi) in 0·05 ml ethanol was added and the mixture incubated at 37°C for 2 h with shaking in air. After addition of carriers (10 μg each of oestrone, oestradiol-17β and oestriol), the reaction was stopped by addition of 5 volumes of acetone. The mixture was homogenized and filtered and the residue washed with acetone. After removal of the acetone, the remaining aqueous phase was extracted with ether (3 × 15 ml) and, after drying ($Na_2SO_4$), the products were separated by thin-layer chromatography on silica gel using chloroform ethyl acetate (9:1, v/v). The [4-$^{14}$C] oestradiol peak, revealed after scanning, was cut out and measured by liquid-scintillation counting. The identity of the oestradiol was confirmed by derivative formation and comparison with authentic compounds in various thin-layer chromatography systems.

*Statistical evaluation.*—Student's two-sided *t* test was applied. Tabulated results are expressed as means ± s.e.

*Liquid-scintillation counting.*—This was carried out using a Triton-toluene phosphor system as described by Li *et al.* (1978). A computerized Packard 2650 instrument was used and quench corrections applied by the external standard method.

### RESULTS

### *Sulphurylation studies*

Formation of APS from ATP and [$^{35}$S] $SO_4^{2-}$ reached a maximum after 20

FIG.—$^{35}$S-Nucleotide synthesis from ATP and [$^{35}$S] $SO_4^{2-}$, using the cytosol fraction from a DMBA-induced rat mammary tumour.

min and then the concentration remained static. This is shown in the Figure and was typical of the data obtained with 4 separate tumours. In Table I, values listed under "PAPS" represent the sum of PAPS + APS, or the "PAPS potential" assayed after 30 min. Oestrogen alone, or oestrogen followed by progesterone, had no significant influence, either on PAPS potential or on the actual PAPS values (not shown).

DHEA sulphotransferase activity was unaltered by hormone administration. In contrast, oestrogen alone, or more especially oestrogen + progesterone, led to an increase in the mean oestrogen sulphotransferase activity, which was 4-fold

TABLE I.—$^{35}$*S-sulphate activation and steroid sulphurylation in mammary tumours*

| Group of rats | Treatment | "PAPS"* (pmol/ 30 min/mg protein) | E₂S† (pmol/h/mg protein) | DHEAS† (pmol/h/mg protein) |
|---|---|---|---|---|
| 1 | Oophorectomized | 358 ± 69 | 18 ± 10 | 43 ± 4 |
| 2 | Oophorectomized + oestradiol-17β | 446 ± 68 | 30 ± 7 | 39 ± 4 |
| 3 | Oophorectomized + oestradiol-17β + progesterone | 362 ± 57 | 78 ± 30 | 32 ± 15 |

* PAPS synthesis measured as the sum of PAPS + adenosine-5′-phosphosulphate (APS). See Fig. 1.
† $E_2S$ = 17β-oestradiol-3-sulphate; DHEAS = DHEA-3-sulphate. Preformed [$^{35}$S]PAPS at saturating evels was used as substrate.
Values are means ± s.e.

TABLE II.—*Metabolism of DHEA and oestrone by mammary tumours*

| | % Conversion of [4-$^{14}$C] DHEA to | | | | | % Conversion of [4-$^{14}$C] oestrone to |
|---|---|---|---|---|---|---|
| Group* | 7α-hydroxy DHEA | 7β-hydroxy DHEA | 7-oxo-DHEA | Total 7-oxygenated DHEA | 5-androstene-3β,17β-diol | oestradiol-17β |
| 1 | 9·52 ± 1·19 | 3·02 ± 0·28 | 2·69 ± 0·39 | 15·3 ± 1·63 | 5·77 ± 0·86 | 4·83 ± 1·27 |
| 2 | 6·91 ± 1·25 | 2·29 ± 0·20 | 0·40 ± 0·31† | 9·64 ± 0·31†† | 5·29 ± 0·87 | 23·6 ± 4·39** |
| 3 | 6·51 ± 1·14 | 3·17 ± 0·37 | 1·92 ± 0·53 | 11·6 ± 1·23††† | 6·81 ± 0·86 | 26·9 ± 0·97*** |

* See Table I.
† Group 2 *vs* Group 1, $P < 0·001$.
†† Group 2 *vs* Group 1, $P < 0·02$.
††† Group 3 *vs* Group 1, $P < 0·1$ (NS). Group 3 *vs* Group 2, $P > 0·25$ (NS).
Separate groups of animals used in experiments with [4-$^{14}$C] oestrone.
** Group 2 *vs* Group 1, $P < 0·005$.
*** Group 3 *vs* Group 1, $P < 0·001$.
Values are means ± s.e.

when progesterone was included. However, owing to the variability between tumours, these results were not significant (Group 3 *vs* Group 1, $P < 0·1$, Table I). Tumours from oophorectomized animals (Group 1) generally showed degenerative regressional changes on histological examination. These changes occurred mostly in the form of pools of mucinous material deposited in the lobular units. In some tumours, the lobular units had lost their distinct architectural form and showed diffuse infiltration of the connective tissue. Tumours from animals receiving oestrogen (Group 2) were generally more cellular, the mucinous material had largely disappeared and infiltration of the connective tissue was common. Oestrogen + progesterone (Group 3) led to tumours with a higher degree of structural organization and an increased secretion of PAS$^+$ material. The stroma of tumours from Groups 1 and 2 showed considerable variegation, and in some cases diffuse inflammatory response with the presence of mast cells.

### *Metabolism of* [4-$^{14}$*C*] *DHEA and* [4-$^{14}$*C*] *oestrone*

Major products of the *in vitro* metabolism of DHEA by rat mammary tumours were previously shown to be 7α- and 7β-hydroxy DHEA, 7-oxo DHEA and 5-androstene-3β,17β-diol (Li *et al.*, 1976). Oestrogen administration to oophorectomized rats led to a drop in each of the 7-oxygenated products of DHEA. Although the total 7-oxygenated products were significantly reduced by some 37% ($P < 0·02$) by oestrogen administration, reduced formation of the main component, 7α-hydroxy DHEA, was not significant ($P < 0·2$). Progesterone tended to reverse the effects of oestrogen, as judged by formation of total 7-oxygenated products (Table II). Formation of 5-androstene-3β,17β-diol was unaltered by hormone administration. Incubation of [4-$^{14}$C] oestrone (3 nmol) with 0·5g samples of tumour minces in Krebs–Ringer buffer fortified with glucose and nicotinamide, caused a 4·8% conversion to oestradiol-17β. Treatment of oophorectomized animals with oestrogen, or oestrogen + progesterone, caused a 5-fold increase in the rate of oestradiol formation from [4-$^{14}$C] oestrone (Table II).

### DISCUSSION

Levels of steroid sulphotransferases in human mammary tumours have been shown to relate to the overall prognosis of the patient, and to the response to hormone ablation (Dao & Libby, 1972). The ability of an individual tumour to generate sufficiently high concentrations of PAPS plays a dominant role in the determination of the degree of sulphurylation of steroids added to the incubations of the tumour extracts (Dao & Libby, 1972; Li *et al.*, 1976; Adams & Chandra, 1977). From data of the type presented in the Figure, it was calculated that the concentration of PAPS in the incubations with DMBA-

tumour cytosol preparations would only reach 1–2 $\mu$M. The $K_m$ for PAPS, using purified bovine placental oestrogen sulphotransferase, is 37 $\mu$M (Adams *et al.*, 1974) and the corresponding values and DHEA sulphotransferase are 13 $\mu$M for enzyme from rat liver and 20 $\mu$M for enzyme from bovine liver (Adams & McDonald, unpublished). Thus, assay of oestrogen and 3$\beta$-hydroxysteroid sulphotransferases were carried out by addition of saturating concentrations of the cosubstrate PAPS. The specific increase in oestrogen sulphotransferase by progesterone (Table I), although not significant in the present experiments, is comparable to a similar influence of this hormone on the enzyme in the endometrium in the gilt (Pack & Brooks, 1974) and human (Buirchell & Hahnel, 1975) uterus. In these cases, the enzyme is induced by progesterone at the end of the proliferative phase of the oestrous and menstrual cycles, and appears to regulate the localized concentration of oestrogen by conversion to the water-soluble inactive conjugate. In the DMBA tumour progesterone may play a similar role, and would provide another example of a "modifying" effect of progesterone on oestrogen action. Although progesterone administration led to large increases in the activity of oestrogen sulphotransferase in some tumours, the effect was not consistent, and the results were not statistically significant. The reason for this is unknown. No apparent differences were noted on histological examination of tumour sections, and rates of tumour regression after oophorectomy were similar, and did not explain the variance in oestrogen sulphotransferase levels.

In mammary tumours from the rat (Li *et al.*, 1976) and human (Couch *et al.*, 1975; Li *et al.*, 1978) 7-oxygenated products are the main metabolites formed from DHEA *in vitro*. It has been established that these conversions are catalysed by enzymes, and are not due to chemical oxidation at the active methylene group at position 7 in 5-androstenes (Li *et al.*, 1976). In an extensive series of experiments, no evidence was found that 7-hydroxy DHEA synthesis represented an intermediate in a more elaborate biosynthetic pathway (Li & Adams, unpublished). However, the presence of the 7-hydroxyl group in 3$\beta$-hydroxy-$\Delta^5$-steroids does convey certain properties which suggest that the 7-hydroxylase may play an important role in the cell. For example, introduction of the 7-hydroxyl group into 5-androstene-3$\beta$,17$\beta$-diol markedly reduced this steroid's capacity to combine with the oestrogen receptor (Li *et al.*, 1978). In addition, the ability of DHEA and 5-androstene-3$\beta$,17$\beta$-diol to inhibit oestrogen sulphotransferase was nullified by 7-hydroxylation (Adams *et al.*, 1978).

Although oestrogen administration did significantly lower formation of 7-oxygenated derivatives of DHEA, the effect was not pronounced. In rat liver, the 7-hydroxylase for DHEA has unusual properties compared to other steroid hydroxylases. It appears to fall more in the class of a "constitutive" enzyme, since it is present in the liver of sexually immature males and females and, in contrast to 16$\alpha$-hydroxylase, the presence of androgen at a critical period of neonatal life is not needed for expression of the enzyme in the liver (Tabei & Heinrichs, 1974). Both 7$\alpha$- and 16$\alpha$-hydroxylases for DHEA in adult male rat liver are sensitive to light/dark modulations, and it has been suggested that the 7$\alpha$-hydroxylase is adrenal-dependent, rather than both gonad- and adrenal-dependent, as is the case of the 16$\alpha$-hydroxylase (Schafer & Colás, 1975). The 17$\beta$-hydrogenation of DHEA to 5-androstene-3$\beta$,17$\beta$-diol was unaffected by hormone administration (Table II) which appears to reflect the situation in rat liver; this transformation being unaffected by castration in either sex (Tabei & Heinrichs, 1974).

Mammary tumours whose growth was stimulated by oestrogen, or oestrogen + progesterone, showed a greater capacity to convert oestrone to oestradiol-17$\beta$ than regressing tumours (Table II). *In vitro* studies have shown that human mammary tumours cultured in the presence of either labelled oestrone or oestradiol-17$\beta$ showed higher ratios of oestradiol-17$\beta$ to oestrone than did normal or benign tissues (Willcox & Thomas, 1972; Geier *et al.*, 1975). The specific activities of oestradiol-17$\beta$ dehydrogenase in subcellular fractions of breast-cancer tissues are lower than those from non-malignant tissue (Pollow *et al.*, 1977). Thus, whilst a decrease in oestradiol-17$\beta$ dehydrogenase could explain the decrease in oestradiol-17$\beta$ metabolism in neoplastic mammary tissue, it is inconsistent with the observed higher conversions of oestrone to oestradiol-17$\beta$. King *et al.* (1965) made the interesting observation that faster-growing DMBA-induced rat mammary tumours converted oestrone to oestradiol at a greater rate than slower-growing tumours. One explanation for the *in vitro* results obtained with DMBA-induced tumours (Table II) and the human tumours referred to above, could be an altered redox state of pyrimidine nucleotides. There is well-documented evidence that oestradiol-17$\beta$ induces glucose-6-phosphate dehydrogenase (GPDH) in both normal and neoplastic mammary tissue (Hilf *et al.*, 1967; Ringler & Hilf, 1975). Furthermore, GPDH activity is higher in neoplastic tissue (Smith *et al.*, 1966; Knox, 1967; Richards & Hilf, 1972) and neoplastic mammary tissue contains higher oestrogen-receptor concentrations than normal mammary tissue, thus suggesting a correlation between GPDH and oestrogen receptor (Daehnfeldt & Schulein, 1975). An increase in $NADPH/NADP^+$, *via* raised GPDH levels, could then account for the increase in the rate of oestradiol-17$\beta$ formation from oestrone in DMBA-induced tumours after oestrogen administration to oophorectomized animals.

The authors are grateful to Dr J. B. Halley for providing the histological data. This work was supported by a grant from the National Health and Medical Research Foundation and the New South Wales State Cancer Council.

### REFERENCES

ADAMS, J. B. (1977) Steroid hormones and human breast cancer: An hypothesis. *Cancer*, **40**, 325.

ADAMS, J. B., ARCHIBALD, L. & CLARKE, C. (1978) Adrenal dehydroepiandrosterone and human mammary cancer. *Cancer Res.*, **38**, 4036.

ADAMS, J. B. & CHANDRA, D. P. (1977) Dehydroepiandrosterone sulfotransferase as a possible shunt for the control of steroid metabolism in human mammary carcinoma. *Cancer Res.*, **37**, 278.

ADAMS, J. B., ELLYARD, R. K. & LOW, J. (1974) Enzymic synthesis of steroid sulphates. IX. Physical and chemical properties of purified oestrogen sulphotransferase from bovine adrenal glands, the nature of its isoenzymic forms and a proposed model to explain its wave-like kinetics. *Biochim. Biophys. Acta*, **370**, 160.

BUIRCHELL, B. J. & HAHNEL, R. (1975) Metabolism of estradiol-17$\beta$ in human endometrium during the menstrual cycle. *J. Steroid Biochem.*, **6**, 1489.

COUCH, R. A. F., SKINNER, S. J. M., TOBLER, C. J. P. & DOUSS, T. W. (1975) The *in vitro* synthesis of 7-hydroxy dehydroepiandrosterone by human mammary tissues. *Steroids*, **26**, 1.

DAEHNFELDT, J. L. & SCHULEIN, M. (1975) High affinity oestradiol receptors and the activity of glucose-6-phosphate dehydrogenase and lactose synthetase in mammary carcinomata of post-menopausal women. *Br. J. Cancer*, **31**, 424.

DAO, T. L. & LIBBY, P. R. (1972) Steroid sulfate formation in human breast tumours and hormone dependency. In *Estrogen Target Tissues and Neoplasia*. Ed. T. L. Dao. Chicago: University of Chicago Press.

GEIER, A., HORN, H., LEVIJ, I. S., LICHTSHTEIN, E. & FINKELSTEIN, M. (1975) The metabolism of $^3$H-estradiol-17$\beta$ in human breast cancer in organ culture. *Eur. J. Cancer*, **11**, 127.

HILF, R., MICHEL, I. & BELL, C. (1967) Biochemical and morphological responses of normal and neoplastic mammary tissue to hormonal treatment. *Recent Progr. Hormone Res.*, **23**, 229.

KING, R. J. B., PANATTONI, M., GORDON, J. & BAKER, R. (1965) The metabolism of steroids by tissue from normal and neoplastic rat breast. *J. Endocrinol.*, **33**, 127.

KNOX, W. E. (1967) The enzyme pattern of neoplastic tissue. *Adv. Cancer Res.*, **10**, 117.

LI, K., ADAMS, J. B. & CHANDRA, D. P. (1976) *In vitro* metabolism of dehydroepiandrosterone by mammary gland and mammary tumours in the rat. *J. Steroid. Biochem.*, **7**, 501.

LI, K., FOO, T. & ADAMS, J. B. (1978) Products of dehydroepiandrosterone metabolism by human mammary tumours and their influence on estradiol receptor binding. *Steroids*, **31**, 113.

MAYNARD, P. V., PIKE, A. W., WESTON, A. & GRIFFITHS, K. (1977) Analysis of dehydroepiandrosterone in human breast tissue using high resolution gas chromatography–mass spectrometry. *Eur. J. Cancer*, **13**, 971.

NICHOLSON, R. I., DAVIES, P. & GRIFFITHS, K. (1978) Interaction of androgens with oestradiol-17$\beta$ receptor proteins in DMBA-induced mammary tumours—a possible oncolytic mechanism. *Eur. J. Cancer*, **14**, 430.

PACK, B. A. & BROOKS, S. (1974) Cyclic activity of estrogen sulfotransferase in the gilt uterus. *Endocrinology*, **95**, 1680.

POLLOW, K., BOQUOI, E., BAUMANN, J., SCHMIDT-GOLLWITZER, M. & POLLOW, B. (1977) Comparison of the *in vitro* conversion of estradiol-17$\beta$ to estrone of normal and neoplastic human breast tissue. *Mol. Cell. Endocrinol.*, **6**, 333.

RICHARDS, A. H. & HILF, R. (1972) Effect of estrogen administration on glucose-6-phosphate dehydrogenase and lactate dehydrogenase isoenzymes in rodent mammary tumors and normal mammary glands. *Cancer Res.*, **32**, 611.

RINGLER, M. B. & HILF, R. (1975) Effect of estrogen on synthesis of glucose-6-phosphate dehydrogenase in R3230 mammary tumours and uteri. *Biochim. Biophys. Acta*, **411**, 50.

SCHAFER, S. J. & COLÁS, A. E. (1975) Influence of adrenalectomy, castration and light cycle on steroid hydroxylase activity in the adult male rat liver. *Endocrinology*, **97**, 1294.

SMITH, J. A., KING, R. J. B., MEGGIT, B. F. & ALLEN, L. N. (1966) Biochemical studies on human and rat breast tissues. *Br. J. Cancer*, **20**, 335.

TABEI, T. & HEINRICHS, W. L. (1974) Enzymatic oxidation and reduction of $C_{19}$-$\Delta^5$-3$\beta$-hydroxy steroids by hepatic microsomes. III. Critical period for the neonatal differentiation of certain mixed-function oxidases. *Endocrinology*, **94**, 97.

WILLCOX, P. A. & THOMAS, G. H. (1972) Oestrogen metabolism in cultured human breast tumours. *Br. J. Cancer*, **26**, 453.